# Smallholders’ coping mechanisms with wheat rust epidemics: Lessons from Ethiopia

**DOI:** 10.1371/journal.pone.0219327

**Published:** 2019-07-31

**Authors:** Moti Jaleta, Dave Hodson, Bekele Abeyo, Chilot Yirga, Olaf Erenstein

**Affiliations:** 1 International Maize and Wheat Improvement Center (CIMMYT), Addis Ababa, Ethiopia; 2 CIMMYT, Texcoco, Mexico; 3 Ethiopian Institute of Agricultural Research (EIAR), Addis Ababa, Ethiopia; International Institute of Tropical Agriculture, NIGERIA

## Abstract

Crops are variously susceptible to biotic stresses–something expected to increase under climate change. In the case of staple crops, this potentially undermines household and national food security. We examine recent wheat rust epidemics and smallholders’ coping mechanisms in Ethiopia as a case study. Wheat is a major food crop in Ethiopia widely grown by smallholders. In 2010/11 a yellow rust epidemic affected over one-third of the national wheat area. Two waves of nationally representative household level panel data collected for the preceding wheat season (2009/10) and three years after (2013/14) the occurrence of the epidemic allow us to analyze the different coping mechanisms farmers used in response. Apart from using fungicides as *ex-post* coping mechanism, increasing wheat area under yellow rust resistant varieties, increasing diversity of wheat varieties grown, or a combination of these strategies were the main *ex-ante* coping mechanisms farmers had taken in reducing the potential effects of rust re-occurrence. Large-scale dis-adoption of highly susceptible varieties and replacement with new, rust resistant varieties was observed subsequent to the 2010/11 epidemic. Multinomial logistic regression models were used to identify the key factors associated with smallholder *ex-ante* coping strategies. Household characteristics, level of specialization in wheat and access to improved wheat seed were the major factors that explained observed choices. There was 29–41% yield advantage in increasing wheat area to the new, resistant varieties even under normal seasons with minimum rust occurrence in the field. Continuous varietal development in responding to emerging new rust races and supporting the deployment of newly released resistant varieties could help smallholders in dealing with rust challenges and maintaining improved yields in the rust-prone environments of Ethiopia. Given the global importance of both wheat and yellow rust and climate change dynamics study findings have relevance to other regions.

## Introduction

Food security is a priority agenda for most developing nations across the world. Though most of these countries strive to attain self-sufficiency in main staple crops, there are several challenges hampering the growth of agricultural production and productivity. Among the many challenges farmers face in crop production, biotic stresses such as plant diseases are a constant menace–and set to increase under climate change with detrimental effects leading to the reduction of food supplies and potential increase in malnutrition [1, 2]. Plant pathogens are estimated to reduce 10–16% of the global harvest each year [3, 4] equivalent to a US$220 billion loss annually [4]. For wheat, annual global losses to pathogens have been estimated at 16–21.5% [5, 6]. Losses solely to wheat yellow rust are estimated to be over 5 million tons annually [7]. An increasing number of studies are indicating shifting distributions of pest and pathogens driven by climate change (e.g., [8]) or forecasting an increasing disease severity [9].

Fungal pathogens that cause wheat rust diseases are a real and evolving threat to food security [10, 11]. Three different wheat rusts are distinguished (stem, yellow and leaf rust), each capable of causing devastating epidemics, but stem and yellow rust typically incur the highest crop losses. Stem rust (caused by the pathogen *Puccinia graminis* f. sp. *tritici*) is the most devastating with complete crop loss possible if conditions are favorable to the disease [12]. Yellow rust (caused by the pathogen *Puccinia striiformis* f. sp. *tritici)* is capable of causing crop losses of >70% under favorable disease conditions [12]. Both pathogens are highly mobile, capable of rapid spread and constantly evolving to produce new races [11, 13].

In this paper, we examine Ethiopia’s recent wheat rust epidemics and smallholders’ coping mechanisms as a case study. Wheat is one of the main staple crops grown in Ethiopia. It provides 13% of the nation’s calorie intake, exceeded only by maize and sorghum [14]. Wheat production and productivity in Ethiopia have shown an increasing trend over the last two decades [15, 16]. Between 2000 and 2016, grain yield increased from 1.16 to 2.68 t/ha whereas the total annual production also increased from 1.2 million to 4.5 million tons [16]. Ethiopia is the largest wheat producer in sub-Saharan Africa [17], but remains a net importer largely due to rapidly rising consumption demands. The Ethiopian government has targeted wheat self-sufficiency by 2023 and the country has huge production potential due to its various favorable agro-ecologies for wheat production [18]. The Ethiopian highlands have long been known as hot spots for wheat rusts [19] and rusts represent the greatest biotic threat to wheat production in Ethiopia. In recent years the frequent occurrence of different wheat rust races, both stem and yellow rust, has increasingly challenged wheat production. A series of major Ethiopian wheat rust epidemics have been documented; yellow rust on the variety *Laketch* in 1977 [20], yellow rust on the variety *Dashen* in 1988 [21], stem rust on the variety *Enkoy* in 1994 [22], yellow rust on *Kubsa* and *Galema* in 2010, and stem rust on *Digalu* in 2013 [23]. Reported yield losses in these epidemics ranged from 50–100% in affected fields. Amongst recent wheat rust challenges, the 2010/11 yellow rust epidemic was the major devastating disease event, affecting over 600,000 ha (more than one-third of Ethiopia’s wheat area) across all major wheat growing agro-ecologies in Ethiopia. Over US$3 million were spent on fungicide and national production losses were estimated to still amount to 15–20%. Associated economic losses were estimated to surpass US$250 million. The epidemic brought Ethiopia’s preceding robust annual wheat productivity growth to an abrupt halt in 2010/11 [15]. Subsequent to the yellow rust epidemic, both government and non-government organizations promoted and distributed yellow rust resistant wheat varieties to replace the most susceptible varieties, starting with the 2011/12 production season.

In addition to varietal attributes related to consumption (farmers’ tastes and preferences), grain yield and rust resistance are the most important criteria Ethiopian farmers consider in their wheat varietal selection [24]. Replacing popular high yielding wheat varieties found to be susceptible to a rust is a particular challenge, as it calls for rust resistant varieties with equivalent or better yield potential and ensuring adequate access for smallholders. An additional challenge is the increasing and constantly evolving rust pressure, which calls for breeding programs and seed systems to ensure a continual flow of new improved resistant varieties that meet farmers’ needs. With diverse attitudes towards risk and differing access to improved varieties for replacement, farmers usually consider different coping mechanisms for a possible recurrence of similar or new rust races. One option is to choose to continue growing their current but susceptible varieties and hope that no rust outbreaks occur; and only if the rust will actually occur in their fields, then possibly take action, e.g. by using (costly) fungicides as *ex-post* coping mechanism. Farmers can also consider *ex-ante* coping mechanisms to reduce the likelihood of rust occurring, e.g. replacing susceptible varieties with resistant ones [25, 26] and/or diversifying the varieties grown to reduce risk [27] and stabilize output [28]. Controlling rust through adopting resistant varieties is considered as the most cost-effective and sustainable long term option [29].

This paper thus takes an important global food staple crop (wheat) and a globally important fungal disease (wheat yellow rust) and uses Ethiopia as a case study to empirically examine smallholders’ *ex-ante* coping mechanisms in the context of recent wheat rust epidemics. Findings from this study are important given the high and evolving rust pressure in Ethiopia and elsewhere, which are set to increase under climate change. The findings have wider implications for biotic stress management through variety dissemination and uptake across developing regions. The remaining part of this paper is structured as follows. Section 2 discusses the conceptual framework and section 3 discusses data and empirical methodology. Analysis results are discussed in section 4 and finally section 5 gives conclusions and implications.

## Conceptual framework

Wheat breeding is a genetic improvement strategy that often revolves around increasing productivity and/or improving and generating resistant varieties to both biotic and abiotic shocks. The two strategies are not independent as tackling (a)biotic shocks contributes to yield stability. In rust-prone environments like Ethiopia, the development and availability of rust resistant wheat varieties may provide a durable rust management strategy. But for farmers to consider varietal replacement when preferred current varieties become susceptible to rusts, there is a need to ensure yields of the new resistant varieties are comparable or better than existing varieties.

Farmers adopt different coping mechanisms to deal with yield shocks–both for experienced (*ex post*) or foreseen (*ex ante*) shocks; whereas others opt to take no action and maintain the status-quo. Some Ethiopian wheat farmers use fungicide control strategies to combat the diseases once diagnosed on their farm (*ex post*), but these are costly (requiring external inputs) and not available to all famers. In anticipation of a foreseen wheat rust threat (*ex ante*), farmers could opt for varietal change to more rust resistant varieties (*varietal resistance*), increase the number of varieties grown (*varietal diversification*) and/or reduce wheat area (*crop substitution*). Past experience and expected occurrence during a given season likely influence such decision-making. Choice of these strategies are also linked with farmers’ attitude towards risk and commercial orientation. More commercial farmers producing wheat mainly for the market are expected to be risk neutral; to be more inclined to varietal resistance aligned with higher productivity aims; and be more informed about varieties and seed sources. Farmers targeting wheat self-sufficiency are expected to be risk avoiding [30] and may prefer varietal diversification where at least the household harvests a given minimum level of wheat production per season under any kind of shock [27]. Farmers may however also opt for crop substitution and switch to other crops less affected by plant diseases, for instance in areas where wheat is less important both as staple or commercial crop; or when the disease occurrence is perceived too challenging and risky and control mechanisms too costly. In general, controlling or reducing the adverse effects of crop diseases on production needs to use combinations of technologies and management practices [31].

## Data and empirical methods

### Data

Data used in this analysis were collected in 2010 and 2014 for the preceding 2009/10 and 2013/14 wheat production seasons, respectively. These two cropping seasons help to assess farmers’ coping mechanisms to the devastating 2010/11 yellow rust epidemic, as the 2009/10 data covers the preceding season and the 2013/14 was the third season after the epidemic. In the 2013/14 survey, sample households were inter alia asked to self-report their experience of the rust epidemic in the 2010/11 season, their expected and actual wheat yield, and their coping strategies. The 2013/14 data allow us to assess whether rust affected farmers reverted to the susceptible but preferred varieties they used to grow, or shifted to the new, resistant varieties, mixed the two coping strategies, or adopted other coping mechanisms like spraying of fungicides where rust occurred.

During the 2009/10 survey, 2096 sample households producing wheat on 3396 plots were surveyed in eight major wheat growing agro-ecologies spread over the major four wheat growing regional states (Oromia, Amhara, SNNPR, and Tigray) constituting around 95% of the national wheat production. In selecting the sample households, we followed stratification by wheat growing agroecologies and eight major wheat growing agroecologies were included. From each agroecology, proportionately, wheat growing districts were randomly selected and in each district four to five wheat growing *kebeles*, the lowest administrative unit in Ethiopia, were randomly selected. In each kebele, 15–18 randomly selected farm households were interviewed. Table 1 shows the distribution of the sample *kebeles* and households by agro-ecological zones and region for the original sample. During the 2013/14 survey, 1921 sample households (from the original 2096 sample households in 2009/10) growing wheat on 3388 wheat plots were re-surveyed. In total, 175 (8%) households from 2009/10 were not interviewed during the 2013/14 survey due to lack of physical presence at home when the enumerators visited their village. Looking at the proportion of the attrition at the agroecological zones (AEZ), i.e., the sampling frame for data collection, the attrition ranges from 6 to 11% by AEZ. Using attrition dummy, we run the mean separation test for key variables (like area under wheat during the 2009/10 season), and we didn’t find significant difference between the two mean values. Thus, we consider that the attrition was random and doesn’t cause much estimation bias.

**Table 1:** Sample *Kebeles* and households (HHs) by agro-ecological zone (AEZ) and region, Ethiopia (original 2009/10 sample).

AEZ	Oromia		Amhara		SNNP		Tigray		Total	
	Kebeles^1^	HHs	Kebeles	HHs	Kebeles	HHs	Kebeles	HHs	Kebeles	HHs
H2	17	295			1	18			18	313
H3	4	66							4	66
M1	2	35	3	36					5	71
M2	21	359	19	339	1	17			41	715
SH1	3	54			2	36			5	90
SA2	2	23							2	23
SH2	10	174	1	18	10	175			21	367
SM2	6	104	14	243			6	104	26	451
**Total**	*65*	*1110*	*37*	*636*	*14*	*246*	*6*	*104*	*122*	*2096*

Note:

^1^Kebele is the lowest administrative unit in Ethiopia.

H2 = Tepid to cool humid mid-highlands, H3 = Cold to very cold humid sub-Afro-Alpine, M1 = Hot to warm moist lowlands, M2 = Tepid to cool moist mid-highlands, SH1 = Hot to warm sub-humid lowlands, SA2 = Tepid to cool semi-arid mid highlands, SH2 = Tepid to cool sub-humid mid highlands, and SM2 = Tepid to cool sub-moist mid highlands.

Thus, we have 1921 panel data at household level to study the coping mechanisms that rust affected households had taken. From these 1921 panel households 1548 (81%) sample households produced wheat during both seasons. The remainder included: 109 that in the end did not produce wheat during both survey seasons, 121 that produced wheat during 2013/14 but not in 2009/10; 143 that produced wheat during 2009/10 but not in 2013/14 season. In both waves of the survey data collection, household and farm characteristics, village characteristics, wheat plot characteristics, wheat varieties and other inputs used at a plot level, wheat production, crop utilization, and respondents’ social network, etc. were documented.

Both the 2009/10 and 2013/14 data collections were done before the establishment of CIMMYT’s Institutional Review Board (ethics committee) in 2016. As such the survey did not get any formal approval of IRB at the time. However, the underlying work followed the prevailing rigorous standards and best practices of international agricultural research at the time. During both surveys, well trained and experienced enumerators speaking the local languages fluently were employed for data collection. Before each interview started with a sample household head (or respondent), the enumerators explained the purpose of the study and the anonymity of all information they provide. Then, enumerators asked respondent’s consent to continue with the interview. All the sample households responded during these two surveys passed through this procedure and provided their full consent orally.

### Empirical model

Farmers select the wheat variety portfolio they grow in a given season *ex ante* (before, or at the beginning of each season), in anticipation of the season’s outlook and considering different attributes of available varieties, including expected yield performance under different (a)biotic stresses. Under normal circumstances, the majority of Ethiopian smallholders would most likely recycle seed of existing varieties for a self-pollinated crop like wheat. Most farmers would thus continue with the varieties at hand, whereas others may decide to switch to new ones or diversify the portfolio of varieties grown (possibly by keeping those at hand and bringing in additional new ones). Such varietal choice decisions likely depend on several factors, among which having experienced a damaging, prior rust epidemic, is likely an important factor favoring varietal replacement in subsequent seasons.

For the analysis, we considered change in the area under resistant varieties and change in the number of wheat varieties grown per season as *ex ante* coping strategies farmers could take to reduce the effect of rust re-occurrence during the season. The changes were obtained by deducting the household level area under resistant varieties and number of varieties grown during the 2009/10 season from the 2013/14 data. To help this computation, yellow rust resistant varieties were identified from the survey data using expert knowledge (Table 2). Compared to the 2009/10 wheat cropping system, a farmer could increase, decrease, or make no change to area under resistant varieties and/or the number of varieties grown during the 2013/14 season. Combining the two (area and variety) with three possible moves (increase, decrease, and no change) could give us nine possible combinations of *ex ante* coping strategies.

**Table 2:** Area share of popular improved wheat varieties from the surveyed plots, Ethiopia.

Major varieties ^*Rust susceptibility*^		2009/10 survey				20013/14 survey			
	Year released	Any seed quality (without considering the extent of seed recycling)		Relatively pure seed (freshly purchased improved seed and not recycled for more than 5 years)		Any seed quality (Without considering the extent of seed recycling)		Relatively pure seed (Freshly purchased improved seed and not recycled for more than 5 years)	
		(ha)	%	(ha)	%	(ha)	%	(ha)	%
Kubsa ^Sy^	1994	395.3	28.9	309.0	22.6	212.8	18.2	196.3	16.8
Galema ^Sy^	1995	119.7	8.7	101.8	7.4	19.7	1.7	16.7	1.4
Tusie ^Ry^	1997	117.6	8.6	104.3	7.6	30.1	2.6	29.1	2.5
Dashen ^Sy^	1984	88.0	6.4	73.2	5.4	37.7	3.2	31.8	2.7
Mada Walabu^Ry^	1999	64.4	4.7	62.4	4.6	16.2	1.4	14.9	1.3
Pavon ^MRy^	1982	42.9	3.1	38.0	2.8	47.7	4.1	46.7	4.0
Digalu ^Ry^	2005	31.3	2.3	30.7	2.2	324.5	27.8	321.5	27.5
ET-13 ^Ry^	1981	23.9	1.7	20.8	1.5	26.8	2.3	23.0	2.0
Enkoy ^Ry^	1974	20.2	1.5	15.6	1.1	4.4	0.4	4.4	0.4
Millennium ^MSy^	2007	4.6	0.3	3.6	0.3	2.3	0.2	2.3	0.2
Danda'a ^MRy^	2010	0.0	0.0	0.0	0.0	67.7	5.8	67.2	5.8
Kakaba ^MRy^	2010	0.0	0.0	0.0	0.0	62.0	5.3	62.0	5.3
Other known improved varieties*		83.3	6.1	78.6	5.7	40.4	3.5	37.1	3.2
Total area under improved vars.		991.3	72.4	838.0	61.2	892.4	76.4	852.9	73.0
Known improved varieties but recycled for >5 seasons		*na*	*na*	153.3	11.2	*na*	*na*	39.5	3.4
Local and unknown varieties		377.7	27.6	377.7	27.6	275.4	23.6	275.4	23.6
Total wheat area (ha)		1369.0	100.0	1369.0	100.0	1167.8	100.0	1167.8	100.0

*Those grown on small area; Rust susceptibility: ^Sy^ Susceptible to yellow rust; ^MSy^ Moderately Susceptible to yellow rust. ^MRy^ Moderately Resistant to yellow rust; ^Ry^ Resistant to yellow rust. na = not applicable

#### Coping mechanism choice

In avoiding or reducing the effect of an uncertain but foreseen production shock, a farmer could choose the best fitting coping mechanism. For a farmer with *J* possible options to respond to an expected rust epidemic shock, the likelihood of choosing one or more combinations of the available options is modelled using multinomial logit (MNL) and given as:

$$P(y=j|X)=\frac{exp(X\beta_{j})}{1+\sum_{h=1}^{J}exp(X\beta_{h})}\;where\;j=0,1,2,\ldots ,J\;and\;h\neq j$$ (1)

Where, *P*(*y* = *j*|*X*) is a response probability that a farmer chooses option *j*, X is a vector of covariates affecting farmer’s response towards the expected but uncertain rust occurrence, and *β* is a vector of parameters to be estimated. The covariates include previous wheat rust and/or disease experience on own farm, household and village characteristics, social networks, and variations in wheat agroecology.

#### Wheat yield implications of coping mechanism choice

To estimate the yield effect associated with coping mechanism choice (either varietal diversification, or varietal resistance, or both) we run a production function that enables comparison of the shifts in the production frontier due to the strategies farmers took in responding to the occurrence of rust in earlier years. Considering the ‘*no change both in area under resistant varieties and number of varieties grown’* strategy as a reference (where farmers kept the same portfolio of wheat varieties grown in 2009/10 in their 2013/14 wheat varietal portfolio), the yield gain (or loss) effects of the above varietal coping mechanisms (alone or in combination) are estimated using different models. First, we used four OLS models with different combination of explanatory variables to see how these coping strategies shifted the yield intercept. Then, we used two-stage least square estimation based on the simultaneous equation specified below (Eq 2). The simultaneity between yield equation and coping strategy choice equation emanates from the assumption that the choice of coping strategy could also be affected by the expected yield a household would like to attain. If this assumption holds true (though to be tested) there is an endogeneity problem where the yield equation could not be estimated using OLS models. In tackling the potential endogeneity, the number of wheat varieties grown during 2010/11 season was considered as an instrumental variable. This variable could affect the possible coping strategy(ies) farmers choose to reduce the effect of rust on their farm but doesn’t have a direct effect on the wheat yield during the 2013/14 production season. The production function specification variously controls for other factors that potentially affect wheat yield such as household and farm characteristics, plot characteristics, and variations in agro-ecology. The production function is given as:

$$\left\{ \begin{array}{l} y_{i}=\alpha_{o}+\beta_{j}S_{ij}+\gamma X_{i}+u_{i}\\ S_{i}=\delta_{0}+\theta y_{i}+\mu Z_{i}+\varepsilon_{i} \end{array}\right.$$ (2)

Where *y*_*i*_ is wheat grain yield of household *i*, and *S*_*i*_ is the coping mechanism followed by household *i*. *X*_*i*_ is a vector of household, plot and agro-ecology related variables affecting wheat yield, and *Z*_*i*_ is household experience of wheat rust and disease occurrence on own farm during the previous seasons. Considering *β*_0_ is the coefficient of *‘no change both in area under resistant varieties and number of varieties grown’* strategy which is used as a reference, any significant *β*_*j*_, where *j* = 1,2,…,8, indicates by how much a household’s wheat yield increased/decreased by choosing the specific coping strategy (changes in varietal diversification, changes in varietal resistance, or any possible combinations of the two). *α*_*o*_ and *δ*_*o*_ are constants. *u*_*i*_ and *ε*_*i*_ are error terms.

Sample farmers were asked to rate soil fertility, slope and soil depth of the wheat plots surveyed during both seasons. Three scales were set for each of these plot characteristics, i.e., 1 = poor/flat/shallow; 2 = medium/gentle/medium; and 3 = good/steep/ deep. As the data analysis was done at household level and most farmers had more than one wheat plot per season, these specific plots were aggregated at household level using area weighted average values that range between one and three.

## Results and discussion

### Descriptive results

**Fig 1:**
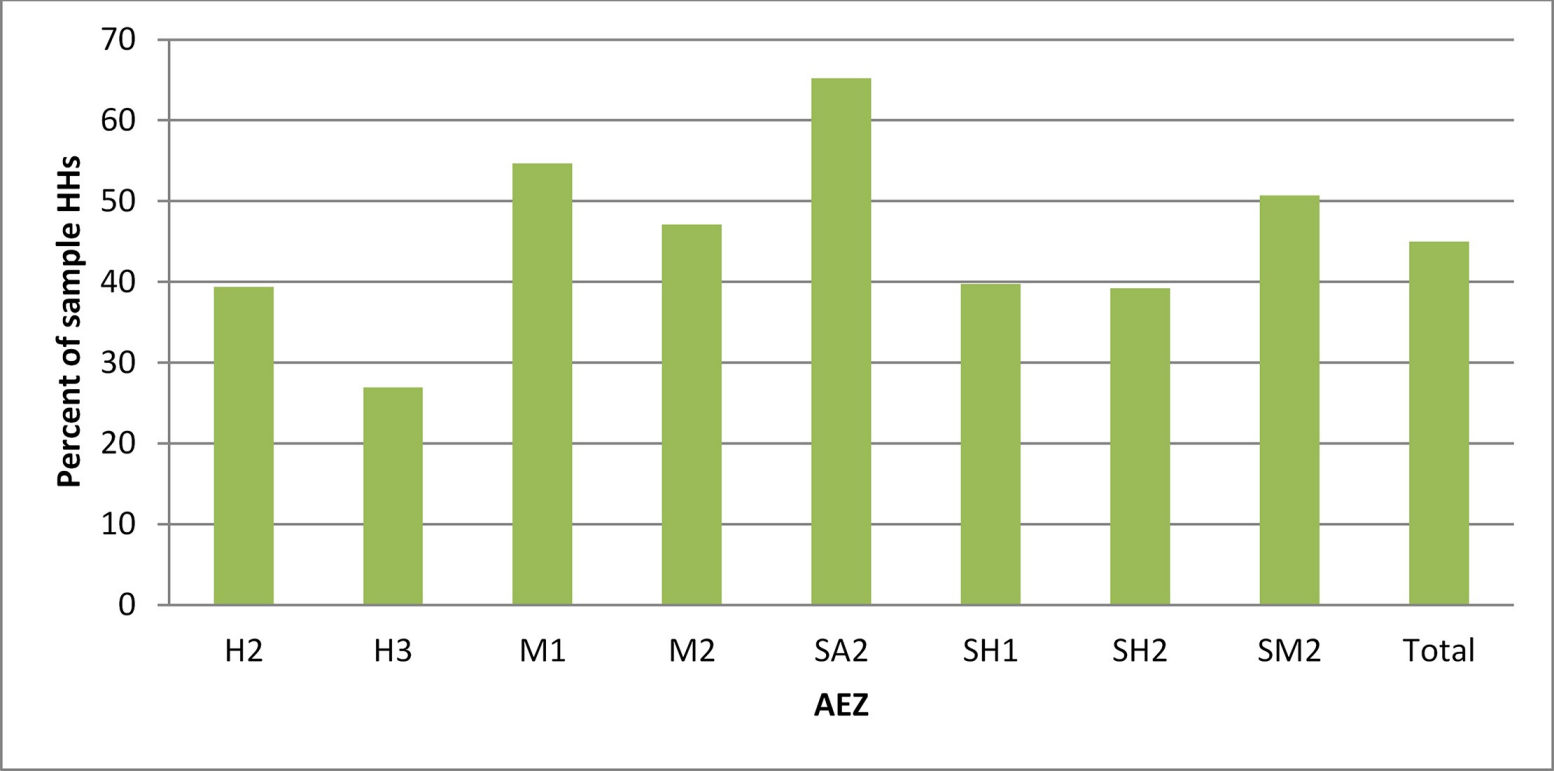
Yellow rust occurrence by agro-ecological zone (AEZ) in 2010/11 production season, Ethiopia (self-reported by sample farmers, 2013/14 survey).

From the 1812 sample households who grew wheat at least in one of the survey seasons, 793 (44%) self-reported the occurrence of yellow rust on their own plots during the 2010/11 epidemic. The epidemic covered all the main wheat agro-ecologies in Ethiopia (Fig 1), being above average in four agro-ecological zones: Hot to warm moist lowlands (M1), Tepid to cool moist mid-highlands (M2), Tepid to cool semi-arid mid highlands (SA2) and Tepid to cool sub-moist mid highlands (SM2).

**Fig 2:**
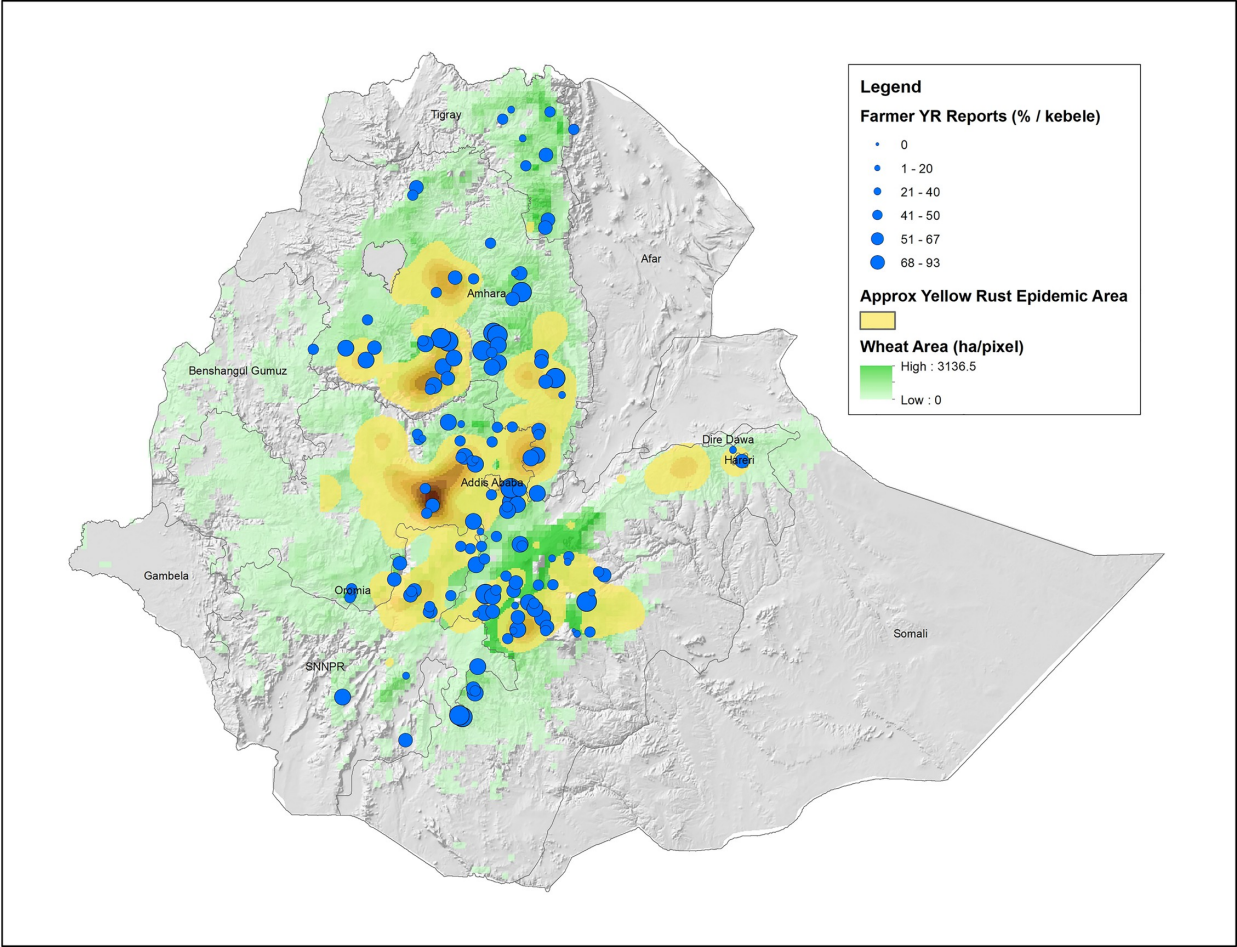
Distribution of farmers reporting yellow rust on-farm (blue dots, scaled by % positive yellow rust reports per kebele) in relation to the approximate area covered by the yellow rust epidemic in 2010 (based on disease survey data).

Fig 2 gives the overlay of wheat potential in the country, the 2010/11 yellow rust severity from disease survey data collected during the season, and the proportion of households reported yellow rust occurrence on their farm from the *kebeles* surveyed under this study. Accordingly, the survey data shows good representation of the yellow rust hotspots.

After the wheat yellow rust epidemic in the 2010/11 cropping season, the government and non-government organizations, seed enterprises and other development supporters increased the supply of resistant varieties (particularly *Digalu*, *Kakaba* and *Danda’a* varieties) to the major rust affected areas. As the two panel surveys were conducted pre- and post the 2010/11 rust epidemic, changes in wheat area allocation to (yellow) rust resistant and susceptible wheat varieties were expected. Based on data from farmers’ report in variety identification during the survey, we discussed with wheat breeders and pathologists working in the national research system and at CIMMYT to categorize these identified varieties into their level of susceptibility to yellow rust. Accordingly, wheat researchers categorized them into susceptible, moderately susceptible, moderately resistant, and resistant varieties to yellow rust. Indeed, wheat area under (yellow) rust susceptible old varieties like *Kubsa*, *Galema*, and *Dashen* had dramatically decreased by the 2013/14 cropping season, whereas area under new resistant varieties such as *Digalu*, *Danda’a* and *Kakaba* had increased substantively (Table 2). *Digalu* was released in 2005, but it was strongly promoted after the 2010/11 yellow rust epidemic. *Kubsa* and *Galema* alone covered 30% of the total surveyed area in the 2009/10 season (for relatively pure seed (new purchase / recycled < 5 years), increasing to 38% for any seed quality–Table 2). This proportion was reduced to 18% of the total surveyed area during the 2013/14 production season (for relatively pure seed, and 20% for any seed quality). Conversely, *Digalu*, only accounted for 2% of the surveyed area in 2009/10, and *Danada’a* and *Kababa* were not grown at all. These three varieties subsequently accounted for 39% of the total area surveyed in the 2013/14 production season (for relatively pure seed, Table 2).

During the 2009/10 survey, sample households were asked to report any wheat stress they had observed in their wheat plots during the specific cropping season. From the 1846 wheat growers identified in the data, about 18% reported stress in wheat production due to disease. Although the cause of the disease was not explicitly asked, it could also be a reason for farmers to opt for new varieties with resistant traits. We included this variable in the list of exogenous variables to explain the variation in household’s coping mechanisms towards the possible re-occurrence of yellow rust during the 2013/14 production season.

From the total 1812 households who grew wheat at least in one of two the survey seasons, 754(42%) increased wheat area under rust resistant varieties during the 2013/14 season (compared to what they had allocated in 2009/10 production season). The level of varietal replacement and remaining share of susceptible varieties are not expected to be uniform among the farm households. Moreover, not all the households directly experienced the yellow rust epidemic on their own farm. Among the 793 farmers who self-reported rust occurrence in 2010/11 in their own wheat plots; 44% increased area under resistant varieties in 2013/14; 47% of the farmers had no change in terms of area under resistant varieties, and 9% of the farmers decreased area under resistant varieties. Among the 1019 farmers who didn’t report rust occurrence in 2010/11, 39% increased the uptake of improved varieties. This shows that farmers’ varietal change due to (a)biotic factors is not always influenced by having experienced a direct negative shock firsthand. Neighbors, extension workers, and other agents through different social networks still can influence them into taking the necessary precautions for any unforeseen future (re-)occurrences. In contrast, farmers who observed rust on their own farm during a given season doesn’t necessarily mean that they change the area under resistant varieties. In Table 3 below, about 47% of those who reported rust during 2009/10 didn’t take any action in increasing area under resistant varieties.

**Table 3:** Changes in area under resistant varieties by experience of rust epidemic, Ethiopia (2013/14 survey).

Change in area under rust resistant varieties (considering 2009/10 survey as a base)	Did rust reportedly occur on wheat plots of sample household during 2010/11?		
	Yes (Column %)	No (Column %)	Total (Column %)
Increased	352 (44%)	402 (39%)	754 (42%)
No change	373 (47%)	488(48%)	861(48%)
Decreased	68 (9%)	129 (13%)	197(11%)
Total	793	1019	1812

Note: For column 3, the percentage doesn’t add to 100%, due to rounding problem.

Table 4 presents the distribution of sample households in terms of the nine coping strategies derived from the adjustments farmers could make in wheat area under resistant varieties and the number of varieties grown each season. Interestingly, there are households in each of the nine combinations of these two strategies. Looking at the distributions closely shows that relatively few farmers went for decreasing area under resistant varieties and increasing the number of varieties grown as a coping strategy. Combination of these two strategies were picked only by 22 farmers. Generally, farmers were tending more towards increasing the area under resistant varieties and either maintaining or decreasing the number of wheat varieties grown each season.

**Table 4:** Distribution of the sample households in their direction of adjustments in area and variety portfolio (for those who grew wheat during both survey seasons).

		Change in area under resistant varieties			Total
		Decrease	No change	Increase	
Change in number of wheat **varieties** grown	Decrease	86	404	258	748
	No change	89	351	304	744
	Increase	22	106	192	320
Total		197	861	754	1812

The 2010/11 yellow rust epidemic was more commonly reported by those households who had relatively larger wheat areas and relatively larger shares of their cereal land under wheat production in the preceding 2009/10 season (Table 5). Overall though, the subsequent changes to the average area allocated to wheat and proportion of wheat area to cereals between the two surveys were similar for those that reported and didn’t report the 2010/11 wheat rust effect on their farm (Table 5).

**Table 5:** Selected indicators of households by reported wheat rust occurrence in 2010/11 season, Ethiopia (2009/10 and 20013/14 surveys).

	HHs reported rust occurrence during 2010/11 *(N = 793)*	HHs didn’t report rust occurrence during 2010/11 *(N = 1019)*	Difference
Wheat area in 2009/10 *(ha/HH)*	0.744	0.677	0.067*
	(0.039)	(0.021)	(0.042)
Change in wheat area *(ha/HH)* (2013/14–2009/10) ^a^	-0.060	-0.066	-0.006
	(0.026)	(0.020)	(0.032)
Proportion of wheat area to cereals in 2009/10	0.482	0.436	0.045***
	(0.010)	(0.009)	(0.013)
Change in the proportion of wheat area to cereals ^a^	-0.004	-0.006	-0.002
	(0.010)	(0.008)	(0.013)
Proportion of wheat area to total operated land in 2009/10	0.357	0.323	0.034***
	(0.008)	(0.007)	(0.011)
Change in the proportion of wheat area to operated land ^a^	-0.020	-0.022	0.002
	(0.008)	(0.007)	(0.011)
Wheat area under resistant varieties in 2013/14 *(ha/HH)*	0.338	0.305	0.033
	(0.024)	(0.017)	(0.029)
Change in area under resistant varieties ^a^	0.182	0.156	0.026
	(0.018)	(0.014)	(0.023)
Change in number of wheat varieties grown per HH ^a^	-0.430	-0.322	-0.108**
	(0.037)	(0.033)	(0.0497)
Change in number of wheat plots per household ^a^	0.024	0.044	0.020
	(0.048)	(0.040)	(0.062)
Change in area allocation to *Kubsa* wheat var. *(ha/HH)* ^a^	-0.105	-0.068	0.037**
	(0.016)	(0.013)	(0.021)

Note: Standard errors are in parentheses

*** and ** are significant at 1% and 5% level, respectively

^a^ Between 2013/14 and 2009/10 surveys.

*Kubsa* and *Galema* were the most predominantly grown varieties in Ethiopia the preceding seasons and during the 2010/11 epidemic season. The major yellow rust resistance gene *Yr27* conferred resistance in both varieties, but its breakdown was key to the 2010/11 epidemic. Large areas planted to these susceptible varieties, the presence of a virulent race of the pathogen and favorable climatic conditions drove the epidemic. The resulting high susceptibility to yellow rust in both varieties caused farmers to seek alternatives and most of the wheat area allocated to the increasingly adopted resistant varieties was at the expense of *Kubsa* and *Galema*. From 554 sample farmers who grew *Kubsa* in the 2009/10 cropping season and were interviewed again during the 2013/14 survey, 293 (53%) of them reported the occurrence of yellow rust on their wheat farm in the 2010/11 cropping season. On aggregate, sample farmers who grew *Kubsa* in 2009/10 reduced wheat area allocated to *Kubsa* during the 2013/14 cropping season, with the area reduction being higher for those farmers who reported rust occurrence during the 2010/11 cropping season (Table 5).

**Fig 3:**
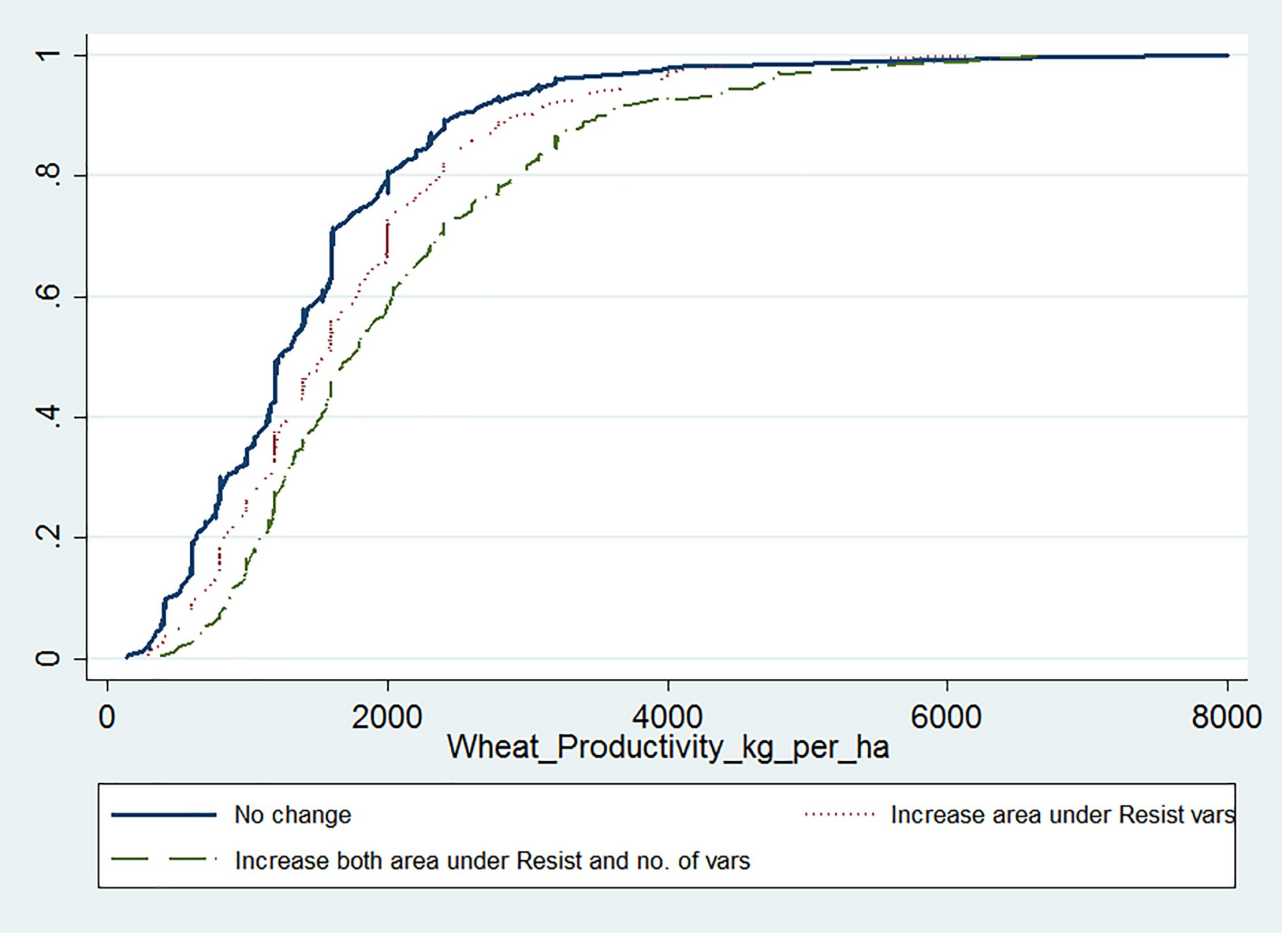
Cumulative distribution of wheat productivity (kg/ha, self-reported) for different wheat rust coping mechanisms, Ethiopia (2013/14 survey).

Wheat yields varied by the rust coping mechanisms in response to the 2010/11 rust epidemic (Table 6). The minimum average yield was observed for those households made no change both in area under resistant varieties and number of varieties grown. On the other hand, maximum average yield was observed for those households who increased both area under resistant varieties and number of varieties grown (Table 6). Similarly, the logistic cumulative yield distribution in Fig 3 shows that households who switched to resistant varieties consistently obtained better wheat yields than those who didn’t change area under resistant varieties and the number of varieties grown.

**Table 6:** Average wheat productivity (kg/ha, self-reported) for different wheat rust coping mechanisms, Ethiopia (2013/14 survey).

		Area under rust resistant varieties		
		Decrease	No change	Increase
No. of varieties grown per season	Decrease	1756.8 *(875*.*3)*	1625.5 *(1004*.*8)*	1762.8 *(937*.*1)*
	No Change	1748.5 (902.5)	1463.8 (1014.9)	1715.2 *(995*.*5)*
	Increase	1753.6 *(1121*.*0)*	2000.6 *(1605*.*7)*	2059.9 *(1200*.*0)*

Note: Standard deviations are in parentheses.

### Empirical results

The Multinomial Logit models assess key factors determining farmers’ decisions in choosing among the nine coping mechanisms stated in Table 6. In the analysis, ‘*no change both in area under resistant varieties and number of wheat varieties grown during each season*’ was considered as a reference to assess the remaining eight combinations of coping strategies. We conducted independence of irrelevant alternatives (IIA) assumption test for the coping strategy options included in the model. Hausman test for the whole sample shows that there is no systemic difference in coefficients when any of the strategic options were dropped.

Model estimation results in Table 7 show that, compared to the base scenario, i.e., the *‘no change’* situation, the likelihood of increasing area under resistant varieties without changing the number of varieties grown per season increases with the education level of household heads. On the other hand, the likelihood of increasing the number of wheat varieties grown without any change in area under resistant varieties decreases at a higher rate for those households growing a more diverse set of wheat varieties during the 2009/10 season. Moreover, educated households and those who were members of cooperatives were more likely to increase both area under resistant varieties and number of varieties grown per season. Those households already using more diversified wheat varieties per season were less likely to choose the combined strategy of increasing both area under resistant varieties and number of varieties grown at the same time.

**Table 7:** Estimated functions explaining wheat rust coping mechanisms use, Ethiopia (Multinomial logit model, whole sample).

Explanatory variables	Increase area under resistant varieties but no change in number of varieties (1)	No change in area under resistant varieties but increased no. of varieties grown (2)	Decrease area under resistant varieties but no change in no. of varieties grown (3)	No change in area under resistant varieties but decreased number of varieties grown (4)	Increase both area under resistant varieties and no. of varieties grown (5)	Decrease area under resistant varieties but increased no. of varieties grown (6)	Decrease both area under resistant varieties and no. of varieties grown (7)	Increase area under resistant varieties but decrease no. of varieties grown (8)
Male household head *(dummy*, *1 = yes)*	-0.496	0.567	-0.982**	-0.608	-0.556	-0.441	-0.989	-0.889*
	(0.342)	(0.813)	(0.454)	(0.513)	(0.439)	(1.123)	(0.758)	(0.536)
Age of household head *(years)*	-0.001	0.016	-0.004	-0.028***	0.003	0.002	-0.044***	-0.011
	(0.007)	(0.014)	(0.011)	(0.010)	(0.010)	(0.023)	(0.015)	(0.011)
Education of household head *(years)*	0.105***	-0.015	0.126***	-0.072*	0.092**	0.148*	0.021	0.077*
	(0.029)	(0.064)	(0.040)	(0.043)	(0.038)	(0.080)	(0.059)	(0.042)
Livestock owned *(tropical livestock unit*, *TLU)*	-0.012	-0.077	-0.021	-0.036	-0.068**	-0.020	-0.098	0.029
	(0.019)	(0.060)	(0.030)	(0.034)	(0.033)	(0.050)	(0.065)	(0.032)
Distance to seed buying point *(walking minutes)*	-0.002	-0.007	0.002	-0.002	0.005	0.014**	0.005	-0.008
	(0.003)	(0.008)	(0.004)	(0.004)	(0.004)	(0.006)	(0.006)	(0.005)
Distance to fertilizer buying point *(walking (minutes)*	-0.001	0.003	-0.004	0.000	-0.009**	-0.013**	-0.003	0.002
	(0.004)	(0.008)	(0.005)	(0.004)	(0.004)	(0.007)	(0.006)	(0.005)
Model farmer *(dummy*, *1 = yes)*	0.031	-0.026	-0.252	-0.261	0.657***	0.109	-1.069***	-0.256
	(0.195)	(0.395)	(0.300)	(0.272)	(0.247)	(0.566)	(0.407)	(0.282)
Number of relatives in the village	-0.001	-0.013	-0.005	0.000	-0.007	-0.016	0.000	-0.005
	(0.003)	(0.011)	(0.005)	(0.003)	(0.005)	(0.013)	(0.005)	(0.006)
Proportion of wheat from area under cereals in 2009	0.522	3.381	1.402	-3.274	2.690	12.526**	1.904	-3.996*
	(1.394)	(2.533)	(2.090)	(2.010)	(1.779)	(5.559)	(3.555)	(2.129)
Square of proportion of wheat from area under cereals in 2009	-0.615	-2.311	0.526	1.267	-0.203	-9.925**	-2.156	2.044
	(1.272)	(2.317)	(1.815)	(1.732)	(1.547)	(5.025)	(2.905)	(1.826)
Area under cereal production 2009	0.208	0.599	0.718***	-0.009	0.619***	0.580**	0.261	0.059
	(0.185)	(0.623)	(0.266)	(0.163)	(0.230)	(0.260)	(0.364)	(0.254)
Square of cereals area in 2009	-0.026	-0.117	-0.050	-0.008	-0.031	-0.008	-0.040	-0.045
	(0.030)	(0.151)	(0.040)	(0.013)	(0.034)	(0.013)	(0.053)	(0.039)
Number of wheat varieties grown in 2009	-1.900	-6.450***	-3.149**	5.280***	-5.051***	0.061	8.849***	5.796***
	(1.284)	(1.268)	(1.352)	(1.450)	(1.250)	(7.675)	(1.920)	(1.465)
Square of no. of wheat varieties grown in 2009	1.189***	1.782***	1.285***	0.316	1.561***	-0.634	-0.192	0.322
	(0.434)	(0.428)	(0.446)	(0.4510	(0.427)	(2.600)	(0.496)	(0.450)
HH observed rust on own farm during 2010/11 season *(dummy*, *1 = yes)*	0.038	-0.143	-0.220	-0.059	0.052	-0.557	-0.914**	0.039
	(0.175)	(0.349)	(0.262)	(0.247)	(0.227)	(0.550)	(0.368)	(0.257)
Whether wheat field in 2009 was affected by disease *(dummy*, *1 = yes)*	0.154	0.154	-0.149	-0.324	0.118	-1.386	-0.741	-0.099
	(0.213)	(0.437)	(0.342)	(0.330)	(0.286)	(1.079)	(0.557)	(0.341)
Number of institutional memberships 2013	-0.051	0.086	0.058	-0.319***	0.009	0.212	-0.273**	-0.249**
	(0.069)	(0.130)	(0.098)	(0.100)	(0.086)	(0.172)	(0.135)	(0.103)
Cooperative membership *(dummy*, *1 = yes)*	0.072	0.409	-0.266	0.097	0.596**	-0.106	-0.035	0.417
	(0.216)	(0.416)	(0.324)	(0.298)	(0.264)	(0.626)	(0.423)	(0.309)
Member of seed group *(dummy*, *1 = yes)*	0.154	-0.179	0.754	0.880	0.287	1.013	0.382	1.020*
	(0.391)	(0.826)	(0.497)	(0.560)	(0.474)	(0.814)	(0.911)	(0.586)
Relative in local admin. *(dummy*, *1 = yes)*	-0.086	-0.937	-1.248*	-0.144	-0.154	-13.972	0.325	0.140
	(0.312)	(0.828)	(0.650)	(0.443)	(0.394)	(558.463)	(0.586)	(0.441)
Member in saving and credit *(dummy*, *1 = yes)*	0.115	0.112	-0.425	0.339	-0.445	0.153	0.013	0.243
	(0.219)	(0.445)	(0.336)	(0.316)	(0.296)	(0.585)	(0.474)	(0.329)
Member in *Equb (dummy*, *1 = yes)*	0.025	0.371	0.380	0.560	0.080	1.004	0.346	-0.816
	(0.333)	(0.635)	(0.445)	(0.490)	(0.438)	(0.761)	(0.711)	(0.568)
*Agro-ecology (dummy*, *H1 = ref*.*)*								
H2	0.912*	1.334	0.432	-0.916	0.101	-0.241	0.680	0.772
	(0.474)	(1.200)	(0.624)	(0.584)	(0.553)	(1.214)	(0.754)	(0.618)
M2	-0.342	0.174	-1.039*	-0.538	-1.050**	-1.095	-0.295	-0.010
	(0.445)	(1.155)	(0.611)	(0.515)	(0.518)	(1.176)	(0.732)	(0.571)
SM2	-0.267	0.869	-0.651	-1.237**	-0.617	0.113	-1.525**	-0.729
	(0.469)	(1.175)	(0.640)	(0.529)	(0.545)	(1.194)	(0.764)	(0.583)
SH1	0.976	0.682	0.669	0.032	0.494	-0.248	0.334	1.567*
	(0.607)	(1.553)	(0.794)	(0.790)	(0.697)	(1.591)	(1.044)	(0.815)
SH2	0.153	0.149	-0.378	-0.674	-1.329**	-16.056	-0.306	0.661
	(0.456)	(1.187)	(0.615)	(0.614)	(0.567)	(760.624)	(0.894)	(0.657)
Constant	0.322	-0.581	0.137	-4.499***	1.193	-6.125	-12.613***	-7.766***
	(1.093)	(1.817)	(1.335)	(1.466)	(1.180)	(5.487)	(2.564)	(1.582)
*Number of observations*	*1*,*544*							
*LR chi2(216)*	*1918*.*3*							
*Prob > chi2*	*0*.*000*							
*Pseudo R2*	*0*.*320*							
*Log likelihood*	*-2042*.*4*							

Note: Standard errors are in parentheses

***, ** and * are significant at 1%, 5% and 10% level, respectively.

More specialized farmers in wheat production (proxied by a higher proportion of wheat area from cereal production) were more likely to increase the number of varieties grown regardless of the changes they made to area under resistant varieties. Households who reported the occurrence of yellow rust on their farm during the 2010/11 season were less likely to adopt the strategy of decreasing both area under resistant varieties and number of wheat varieties grown per season. These households were the most affected by the 2010/11 yellow rust epidemic and understood the consequence of decreasing area allocated to resistant varieties and the effect of varietal level specialization when disease occurs. Regardless of changes made to area under resistant varieties, famers with more social networks (proxied by institutional memberships) were less likely to adopt a decreasing the number of wheat varieties as a coping mechanism for potential re-occurrence of wheat rust.

The 2010/11 rust epidemic observed on own farm and associated yield loss estimates could be the potential driver for farmers to change wheat area under resistant varieties and/or the number of varieties grown as a coping mechanism for a possible rust re-occurrence. It is expected that those farmers severely affected by the 2010/11 rust epidemic (measured by the estimated yield loss) would respond aggressively towards making more changes in area and diversity of wheat varieties grown. The estimated yield loss due to rust was reported only by those households who mentioned rust occurrence on their farm during the 2010/11 cropping season. Thus, the effect of estimated yield loss on the intensities of coping mechanisms is assessed only for these sub-samples. We regressed the magnitude of changes made to area under resistant varieties and change in the number of wheat varieties grown per season (between 2009/10 and 2013/14 seasons) on a set of explanatory variables including the estimated yield loss.

Results in Table 8 shows that the estimated yield loss affected the change in area under resistant varieties positively but, at a decreasing rate for larger estimated losses. Better educated and male household heads, model farmers, households who owned more livestock, and those who were members to cooperatives increased their wheat area under rust resistant varieties. This might imply that better-off farmers might have had more privileges to access rust resistant varieties compared to their counterfactuals. In terms of agroecology, relatively, households in H2 (Tepid to cool humid mid-highlands), and SH2 (Tepid to cool sub-humid mid highlands) increased their wheat area under resistant varieties. These zones are highly suitable for rust development due to their favorable temperatures and humid environment [32]. On the other hand, change in the number of wheat varieties grown per household decreased with wheat dominance in the portfolio of cereals grown.

**Table 8:** Explaining variations in changes made to wheat area under resistant varieties and number of varieties grown as a coping strategy (between 2009/10 and 2013/14 seasons).

Explanatory variables	Change in area under resistant varieties (in ha)	Change in the number of wheat varieties grown per season
	Coeff. (Std.Err)	Coeff. (Std.Err)
Yield loss estimate due to rust in 2010/11 *(t/ha)*	0.141**	0.108
	(0.055)	(0.112)
Square of yield loss due to rust in 2010/11 *(t/ha)*^*2*^	-0.027**	-0.017
	(0.012)	(0.025)
Male household head *(dummy*, *1 = yes)*	0.134	-0.023
	(0.083)	(0.168)
Age of household head *(years)*	0.001	0.005
	(0.002)	(0.003)
Education of household head *(years)*	0.012**	0.007
	(0.006)	(0.012)
Livestock owned *(tropical livestock unit*, *TLU)*	0.011**	0.012
	(0.005)	(0.010)
Distance to seed buying point *(walking minutes)*	-0.001	0.002
	(0.001)	(0.002)
Distance to fertilizer buying point *(walking minutes)*	0.001	-0.002
	(0.001)	(0.001)
Model farmer *(dummy*, *1 = yes)*	0.135***	-0.141*
	(0.041)	(0.083)
Number of relatives in the village	0.000	0.001
	(0.001)	(0.001)
Proportion of wheat from area under cereals in 2009	-0.104	-0.413***
	(0.076)	(0.149)
Area under cereal production during 2009	-0.140***	-0.076***
	(0.013)	(0.025)
Number of wheat varieties grown in 2009	0.119***	
	(0.022)	
Whether wheat field in 2009/10 was affected by disease *(dummy*, *1 = yes)*	0.004	0.086
	(0.047)	(0.095)
Number of institutional memberships 2013	-0.011	0.028
	(0.015)	(0.031)
Cooperative membership *(dummy*, *1 = yes)*	0.127***	-0.150
	(0.046)	(0.092)
Member of seed group *(dummy*, *1 = yes)*	0.012	-0.291
	(0.092)	(0.187)
Relative in local admin. *(dummy*, *1 = yes)*	0.058	-0.227*
	(0.067)	(0.136)
Member in saving and credit *(dummy*, *1 = yes)*	-0.004	-0.049
	(0.050)	(0.100)
Member in *Equb (dummy*, *1 = yes)*	-0.099	0.057
	(0.074)	(0.151)
*Agro-ecology (dummy*, *H1 = ref*.*)*		
H2	0.327***	0.468***
	(0.086)	(0.174)
M2	-0.004	0.440***
	(0.077)	(0.155)
SM2	0.019	0.201
	(0.078)	(0.158)
SH1	0.185	0.561**
	(0.112)	(0.227)
SH2	0.206**	0.286
	(0.087)	(0.175)
Constant	-0.251	-0.821***
	(0.155)	(0.311)
*Number of obs*.	*693*	*693*
*F(k*, *n-k)*	*8*.*25*	*2*.*39*
*Prob*. *> F*	*0*.*000*	*0*.*000*
*R-squared*	*0*.*236*	*0*.*079*
*Adj*. *R-squared*	*0*.*208*	*0*.*046*

Note

***. **, and * are significant at 1%, 5%, and 10% levels, respectively.

To explore the extent of wheat yield gain (or loss) associated with different coping mechanisms used, we run four OLS regressions and a two-stage least square (2SLS) estimation (taking *‘no change both in area under resistant varieties and number of varieties grown per season’* as a reference). The basic Model (1) includes only the coping mechanisms, and additional models increasingly control for the effect of other factors affecting yields at plot and household levels. In Model (2) we added average input use and plot characteristics aggregated at a household level using area-weighted average scores reported by farmers for soil fertility, slope, and soil depth. In addition, subsequently, we added household characteristics in Model (3) and agro-ecology dummies in Model (4). Model (5) gives the 2SLS estimation results derived from the simultaneous equation stated in Eq (2). We conducted endogeneity tests for the simultaneous equation specified. Test results showed that there is a week endogeneity of the coping mechanisms in the yield estimation [F(8,1484) = 1.83, Prob>F = 0.0672]. Thus, we used the predicted probabilities of the coping strategies obtained from the first stage MNL estimation in the second stage yield estimation (Model 5). It is worth noting that coefficient estimates of the coping strategies included in Model (5) are not interpreted directly as these variables are the predicted probabilities derived from the first stage MNL estimation.

Wheat grain yield increases were observed for those who increased both area under resistant varieties and number of wheat varieties grown per season. Results in Models (1) to (4) (Table 9) show that the average wheat grain yield increment for those households increased both area under resistant varieties and the number of wheat varieties grown per season ranges between 420 to596 kg/ha. This is 29–41% higher than the average wheat yield attained by those households who made no change in terms of area under resistant varieties and number of varieties grown per season. Compared to the ‘no change’ strategy, increasing varietal diversity while decreasing area under resistant varieties had no significant yield effect across models. On average, the use of resistant varieties thus provided important yield benefits, in addition to conferring (yellow) rust resistance and enhancing yield stability in the event of a (yellow) rust epidemic. The additional yield gain justifies the quick expansion of yellow rust resistant varieties observed after the 2010/11 epidemic and calls for continuous support to the research and extension programs to develop and disseminate improved wheat varieties with resistant traits to old and newly emerging rust races.

**Table 9:** Estimated functions explaining wheat grain yield in relation to different wheat rust coping mechanisms and other explanatory factors, Ethiopia (OLS, self-reported wheat yields, kg/ha—2013/14–2009/10).

Explanatory variables	Model (1) OLS	Model (2) OLS	Model (3) OLS	Model (4) OLS	Model (5) 2SLS
*Coping mechanism*^*1*^					
Increased area under YRR ^a^ but no change in no. of variety *(1 = yes)*	251.3***	114.1	68.0	16.5	-343.4^b^
	(83.4)	(78.1)	(78.0)	(77.9)	(436.7)
No change in area under YRR but increased no. of varieties *(1 = yes)*	536.8***	581.9**	608.4***	585.4***	-1056.8** ^b^
	(120.2)	(110.7)	(109.8)	(108.3)	(407.3)
Decreased area under YRR but no change in no. of varieties *(1 = yes)*	284.6**	294.7**	237.1**	171.8	47.7 ^b^
	(126.9)	(117.0)	(116.6)	(115.8)	(580.4)
No change in area under YRR but decreased no. of varieties *(1 = yes)*	161.7**	142.2*	145.5*	135.2*	29.9 ^b^
	(85.5)	(79.6)	(79.2)	(79.2)	(180.9)
Increased both area under YRR and no. of varieties *(1 = yes)*	596.1***	528.3***	484.1***	420.1***	753.4** ^b^
	(96.2)	(92.3)	(91.9)	(92.0)	(368.1)
Decreased area under YRR but increased no. of varieties *(1 = yes)*	289.7	227.5	165.5	86.5	541.5 ^b^
	(232.5)	(216.2)	(214.5)	(212.1)	(606.4)
Decreased both area under YRR and no. of varieties *(1 = yes)*	293.0**	296.5**	259.8	168.6	411.2 ^b^
	(142.1)	(131.0)	(130.4)	(130.0)	(437.6)
Increased area under YRR but decreased no. of varieties *(1 = yes)*	299.0***	214.7**	180.7**	153.2*	83.0 ^b^
	(87.6)	(82.9)	(82.4)	(81.9)	(262.6)
Average quantity of fertilizer used for wheat *(kg/ha)*		2.9***	2.8***	3.0***	3.1***
		(0.3)	(0.3)	(0.3)	(0.3)
Average quantity of herbicide used for wheat *(kg/ha)*		136.1***	128.9***	118.5***	132.6***
		(22.4)	(22.3)	(22.4)	(20.9)
Average quantity of pesticide used for wheat *(kg/ha)*		373.2**	331.9**	285.8*	327.8**
		(167.2)	(165.8)	(164.6)	(151.5)
Wheat affected by stress *(dummy*, *1 = yes)*		-405.9***	-390.8***	-380.3***	388.4***
		(55.8)	(55.6)	(55.2)	(52.3)
Wheat affected by water logging *(dummy*, *1 = yes)*		-264.0***	-250.0**	-300.3***	-274.9***
		(101.2)	(100.6)	(99.8)	(94.8)
Wheat affected by frost *(dummy*, *1 = yes)*		-248.9***	-250.8***	-289.0***	-243.8***
		(92.3)	(91.9)	(92.4)	(87.3)
Soil fertility *(1 = poor*, *……*.*3 = good)*		117.2**	107.1**	98.9**	53.7
		(45.7)	(45.4)	(45.3)	(43.7)
Slope *(1 = flat*, *……*.*3 = steep)*		-158.0***	-147.3***	-141.3***	-175.1***
		(45.4)	(45.3)	(45.1)	(43.1)
Soil depth *(1 = shallow*, *……3 = deep*		63.7	67.9	73.9*	72.5*
		(43.9)	(43.8)	(43.4)	(41.7)
*Household Characteristics*					
Male household head *(dummy*, *1 = yes)*			-74.1	-66.5	-8.9
			(96.0)	(94.7)	(97.2)
Age of household head *(years)*			-5.1**	-5.8***	-4.8**
			(2.1)	(2.0)	(2.0)
Education of household head *(years)*			31.8***	28.8***	24.5**
			(8.0)	(8.0)	(10.7)
Household head is model farmer *(dummy*, *1 = Yes)*			115.6**	118.0**	96.1*
			(53.6)	(53.3)	(54.9)
Walking minutes to fertilizer buying center			-0.1	-0.3	-0.3
			(0.5)	(0.6)	(0.5)
Livestock owned *(TLU)*			-1.5	-3.0	-4.4
			(5.9)	(5.8)	(5.7)
*Agro-ecology (dummy*, *H1 = ref*.*)*					
H2				-105.9	-88.7
				(223.8)	(222.3)
3				481.2*	167.3
				(280.3)	(276.0)
M1				-435.4*	-498.7**
				(256.0)	(240.8)
M2				-446.0**	-481.1**
				(221.1)	(209.5)
SM2				-322.0	-346.5
				(223.5)	(212.1)
SH1				-625.7***	-632.1***
				(239.9)	(232.3)
SH2				-423.6*	-420.8*
				(224.5)	(215.7)
Constant	1463.8***	1082.2***	1294.3***	1689.7***	1416.2***
	(57.2)	(87.0)	(166.8)	(280.4)	(312.2)
*Number of obs*	*1*,*638*	*1*,*638*	*1*,*636*	*1*,*636*	*1*,*523*
*F-Value*	*6*.*150*	*21*.*91*	*18*.*19*	*16*.*17*	*17*.*36*
*Prob > F*	*0*.*000*	*0*.*000*	*0*.*000*	*0*.*000*	*0*.*00*
*R-squared*	*0*.*029*	*0*.*187*	*0*.*206*	*0*.*232*	*0*.*259*
*Adj R-squared*	*0*.*025*	*0*.*178*	*0*.*195*	*0*.*218*	*0*.*244*

Note

^a^ YRR = Yellow Rust Resistant; Standard errors are in parentheses

^b^Coefficients are estimated based on predicted probability variables derived from multinomial logit estimation in stage 1. Thus, estimates are not interpreted as a simple intercept shift.

^1^reference is ‘No change both in area under resistant varieties and number of varieties grown per season’

***. **, and * are significant at 1%, 5%, and 10% levels, respectively.

We can conservatively estimate the benefits of yellow rust resistance in Ethiopia considering the 2013/14 national wheat area (1.6 million ha), the average farm-gate wheat price ($350 per ton), the 26% wheat area increment to yellow rust resistant varieties from this study, and the 29–41% yield gain due to increasing both area under yellow rust resistant varieties and increasing number of varieties grown per season (Table 9). We thus estimate that wheat growing smallholders in Ethiopia obtained an additional income of US$61–87 million due to enhancing their use of yellow rust resistant wheat varieties during the 2013/14 production season.

### Conclusions and implications

Crop disease epidemics, typified by wheat rusts, result in large scale crop losses that put farmers’ income and food security at risk. The problem is even more challenging in smallholder farming systems like in Ethiopia with millions of households producing wheat as a major staple and cash crop. Thus, facing the possible incidence of a rust epidemic, farmers need to decide whether to take necessary rust coping mechanisms to avoid or at least reduce potential damage.

Farmers’ response to the 2010/11 wheat yellow rust shock in structurally shifting or increasing the use of rust resistant wheat varieties in the subsequent production seasons were remarkable. Even under the relatively less rust affected seasons like 2013/14 covered by the survey, average wheat grain yields were relatively higher for households who had shifted to resistant varieties. As grain yield is usually the top priority of farmers in varietal selection, such yield performance of the resistant varieties under normal seasons encouraged farmers to switch from extremely susceptible but high yielding varieties like *Kubsa* to recently promoted resistant varieties such as *Digalu*, *Kakaba*, *Danda’a* and others. The benefits to farmers, in terms of increased productivity and economic gain, through varietal replacement with new, improved rust resistant varieties was clearly demonstrated.

In supporting wheat dependent smallholders to withstand the recurrent rust challenges, continuous wheat breeding is essential in generating new resistant varieties that respond to the ever-changing rust races over time. The challenges in rust-prone environments like Ethiopia are considerable. A variety considered as resistant to a specific rust race can become susceptible to a new race, especially if resistance is based on a single major gene. A case in point is *Digalu*, resistant to prevailing yellow and stem rust races in Ethiopia in 2010/11. *Digalu* was extensively multiplied and distributed to farmers with the aim of replacing *Kubsa* and *Galama* that were devastated by yellow rust in 2010/11. However, in the 2013/14 production season, *Digalu* itself was severely affected by a new stem rust race TKTTF [23] and subsequently in 2016/17 *Digalu* became highly susceptible to a new yellow rust race PstS11 [17]. Recent experience in the rust-prone wheat systems of Ethiopia has shown that reliance on one or two resistant varieties and keeping the wheat landscape to a low level of varietal diversification (or more critically, low rust resistance gene diversity) represents a threat if these few varieties become susceptible to newly emerging rust races. In this regard, wheat breeding, seed multiplication, distribution and extension services need to work in a better synergy and provide up-to-date information to one another on the performance of the different wheat varieties in farmers’ field and the candidate and newly released varieties in the research system. Ultimately, the long-term goal should be widespread cultivation of a diverse range of multiple rust resistant varieties with polygenic (multiple minor genes in combination with multiple major genes), race non-specific resistance and a supporting robust variety development and delivery pipeline. We also recommend further study to explore optimal combination level of land allocation to rust resistant varieties and use of new rust resistant varieties that could give maximum yield without necessarily rationing out other subsistence crops.

## Supporting information

S1 Fig Area shares of different wheat varieties during 2009/10 and 2013/14 seasons, Ethiopia (survey data). (TIF)

## References

[1] Rosenzweig C, Iglesias A, Yank XB, Epstein P, Chivian R. Climate change and extreme weather events: Implications for food production, plant diseases, and pests. Global Challenge and Human Health. 2001;2: 90–104.

[2] Gregory PJ, Johnson SN, Newton AC, Ingram JSI. Integrating pests and pathogens into the climate change/ food security debate. J Exp Bot. 2009;60: 2827–2838. 10.1093/jxb/erp080 19380424

[3] Strange RN, Scott PR. Plant disease: a threat to global food security. Ann Rev Phytopathol. 2005;43: 83–116. 16078878 10.1146/annurev.phyto.43.113004.133839

[4] Chakraborty S, Newton AC. Climate change, plant diseases and food security: an overview. Plant Pathol. 2011;60: 2–14.

[5] Oerke EC. Crop losses to pests. J Agr Sci. 2006;144: 31–43.

[6] Savary S, Willocquet L, Pethybridge SJ, Esker PD, McRoberts N, Nelson AD. The global burden of pathogens and pests on major food crops. Nature Ecology & Evolution. 2019;3: 430–439. 30718852 10.1038/s41559-018-0793-y

[7] Beddow JM, Pardey PG, Chai Y, Hurley TM, Kriticos DJ, Braun H-J, et al Research investment implications of shifts in the global geography of wheat stripe rust. Nat Plants. 2015;1: 15132 10.1038/nplants.2015.132 27251389

[8] Bebber DP, Ramotowski MAT, Gurr SJ. Crop pests and pathogens move polewards in a warming world. Nat Clim Change. 2013;3: 985–988.

[9] Caubel J, Launay M, Ripoche D, Gouache D, Buis S, Huard F, et al (2017) Climate change effects of leaf rust of wheat: Implementing a coupled crop-disease model in a French regional application. Eur J Agron. 2017;90: 53–66.

[10] Chaves MS, Martinelli JA, Wesp-Guterres C, Graichen FAS, Brammer SP, Scagliusi SM, et al (2013) The importance of food security of maintaining rust resistance in Wheat. Food Secur. 2013;5: 157–1179.

[11] Singh RP, Singh PK, Rutkoski J, Hodson DP, He X, Jorgensen LN, et al Disease impact on wheat yield potential and prospects of genetic control. Annu Rev Phytopathol. 2016;54: 303–322. 10.1146/annurev-phyto-080615-095835 27296137

[12] Vergara-Diaz O, Kefauver SC, Elazab A, Nieto-Taladriz MT, Araus JL. Grain yield loss in yellow-rusted durum wheat estimated using digital and conventional parameters under field conditions. The Crop J. 2015;3: 200–210.

[13] Ali S, Rodriguez-Algaba J, Thach T, Sorensen CK, Hansen JG, Lassen P, et al Yellow rust epidemics worldwide were caused by pathogen races from divergent genetic lineages. Front Plant Sci. 2017; 8 (article 1057): 1–14. 10.3389/fpls.2017.00001 28676811 PMC5477562

[14] Berhane G, Paulos Z, Tafere K, Tamru S. Food Grain Consumption and Calorie Intake Patterns in Ethiopia. ESSP II Working Paper No. 23, 2011 May. http://ebrary.ifpri.org/cdm/ref/collection/p15738coll2/id/124853. Cited 03 July 2019.

[15] Central Statistical Agency (CSA). Report on area and production of major crops. Statistical Bulletin, April 2011, Addis Ababa.

[16] Central Statistical Agency (CSA). Report on area and production of major crops. Statistical Bulletin 584, 2017. Addis Ababa.

[17] Food and Agriculture Organization of the United Nations (FAO). Food Chain Crisis Early Warning Bulletin: Forecasting threats to the food chain affecting food security in countries and regions, No. 28. (July-Sept. 2018). http://www.fao.org/3/ca0354en/CA0354EN.PDF Cited 03 July 2019.

[18] Negassa A, Shiferaw B, Koo J, Sonder K, Smale M, Braun HJ, et al The Potential for Wheat Production in Africa: Analysis of Biophysical Suitability and Economic Profitability. Mexico, D.F.: CIMMYT, 2013 https://repository.cimmyt.org/handle/10883/4015 Cited 03 July 2019.

[19] Saari EE, Prescott JM. World distribution in relation to economic losses In: Roelfs AP, and Bushnell WR, editors. The Cereal Rusts Vol. II; Diseases, Distribution, Epidemiology, and Control. Orlando: Academic Press; 1985 pp.259–298.

[20] Hulluka M, Woldeab G, Andnew Y, Desta R, Badebo A. Wheat pathology research in Ethiopia In: Gebre-Mariam H, Tanner DG, Hulluka M, editors. Wheat Research in Ethiopia. Addis Ababa: A historical perspective, IAR/CIMMYT; 1991 pp.173–217.

[21] Badebo A, Stubbs RW, van Ginkel M, Gebeyehu G. Identification of resistance genes to Puccinia striiformis in seedlings of Ethiopian and CIMMYT bread wheat lines. Neth J Plant Pathol. 1990;96: 199–210.

[22] Shank R. Wheat stem rust and drought effects on Bale agricultural production and future prospects. Report on February 17–28 assessment. United Nations Emergencies Unit for Ethiopia. Addis Ababa. 1994. http://www.africa.upenn.edu/eue_web/s_rust94.htm Cited 03 July 2019.

[23] Olivera P, Newcomb M, Szabo LJ, Rouse M, Johnson J, Gale S, et al Phenotypic and genotypic characterization of race TKTTF of *Puccinia graminis* f. sp. *tritici* that caused a wheat stem rust epidemic in southern Ethiopia in 2013–14. Phytopathol. 2015;105: 917–28. 10.1094/PHYTO-11-14-0302-FI 25775107

[24] Bishaw Z, Alemu D. Farmers’ perceptions on improved bread wheat varieties and formal seed supply in Ethiopia. Int J Plant Prod. 2017;11: 117–130.

[25] Joshi AK, Azab M, Mosaad M, Moselhy M, Osmanzai M, Gelalcha S, et al Delivering rust resistant wheat to farmers: A step towards increased food security. Euphytica. 2011;179: 187–196.

[26] Singh RP, Hodson DP, Jin Y, Lagudah ES, Ayliffe MA, Bhavani S, et al Emergence and Spread of New Races of Wheat Stem Rust Fungus: Continued Threat to Food Security and Prospects of Genetic Control. Phytopathol. 2015;105: 872–884. 10.1094/PHYTO-01-15-0030-FI 26120730

[27] Di Falco S, Chavas JP. On crop biodiversity, risk exposure, and food security in the highlands of Ethiopia. Am J Ag Econ. 2009;91: 599–611.

[28] Suresh A, Praveen KV, Reddy AA, Singh DR. Risks in rainfed agriculture and farmers’ adaptation practices: A case of cotton farmers of Maharashtra. Ind J Ag Econ. 2017;72: 362–374.

[29] Hodson DP. Shifting boundaries: challenges for rust monitoring. Euphytica. 2011;179: 93–104.

[30] Fafchamps M. Cash crop production, food price volatility, and rural market integration in the Third World. Am J Ag Econ. 1992;74(1): 90–99.

[31] Krupinsky JM, Bailey KL, McMullen MP, Gossen BD, Turkington TK. Managing plant disease risk in diversified cropping systems. Agron J. 2002;94: 198–209.

[32] Tolemariam A, Jaleta M, Hodson D, Alemayehu Y, Yirga C, Abeyo B. Wheat Varietal Change and Adoption of Rust Resistant Wheat Varieties in Ethiopia from 2009/10 to 2013/14. Socioeconomics Program Working Paper 12, Mexico, CDMX: CIMMYT. 2018. https://repository.cimmyt.org/handle/10883/19532?locale-attribute=en. Cited 03 July 2019.

