# Supplementary figures and images for: Smallholders’ coping mechanisms with wheat rust epidemics: Lessons from Ethiopia
Source: PLoS One. 2019 Jul 31;14(7):e0219327. doi: 10.1371/journal.pone.0219327 (PMC6668782; doi:10.1371/journal.pone.0219327)

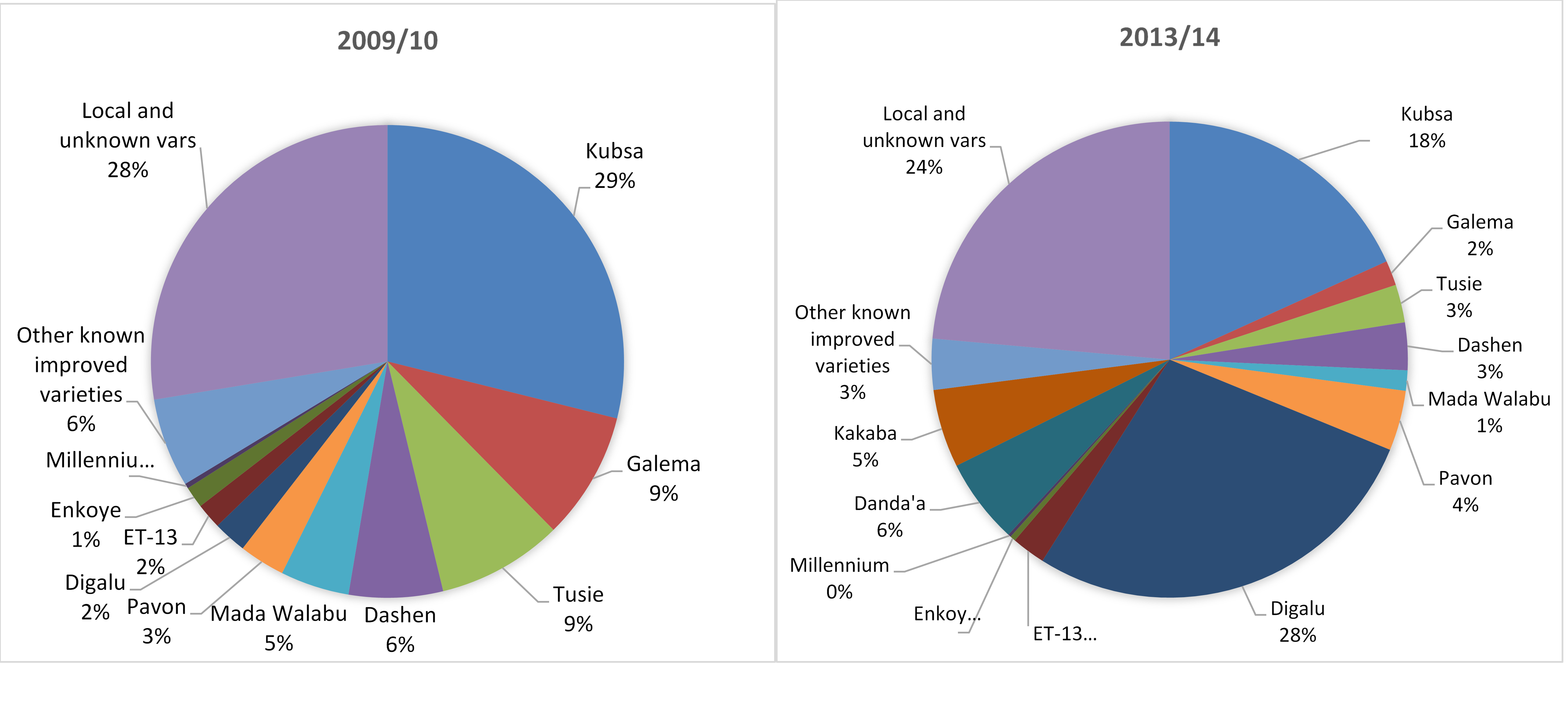

Supplement: S1 Fig — (TIF) [file pone.0219327.s001.tif]
